# Contribution to the Study of the Relation between Microstructure and Electrochemical Behavior of Iron-Based FeCoC Ternary Alloys

**DOI:** 10.1155/2012/798043

**Published:** 2012-01-12

**Authors:** Farida Benhalla-Haddad, Sif Eddine Amara, Abdelkader Benchettara, Kamel Taibi, Rafika Kesri

**Affiliations:** ^1^Laboratory of Electrochemistry, Corrosion, Metallurgy and Inorganic Chemistry, Faculty of Chemistry, University of Science and Technology Houari Boumediene, P.O. Box 32, El-Alia, Bab Ezzouar, Algiers 16111, Algeria; ^2^Laboratory of Materials Science and Engineering, University of Science and Technology Houari Boumediene, P.O. Box 32, El-Alia, Bab Ezzouar, Algiers 16111, Algeria

## Abstract

This work deals with the relation between microstructure and electrochemical behavior of four iron-based FeCoC ternary alloys. First, the arc-melted studied alloys were characterized using differential thermal analyses and scanning electron microscopy. The established solidification sequences of these alloys show the presence of two primary crystallization phases (*δ*(Fe) and graphite) as well as two univariante lines : peritectic L + *δ*(Fe)↔*γ*(Fe) and eutectic L↔*γ*(Fe) + C_graphite_. The ternary alloys were thereafter studied in nondeaerated solution of 10^−3^ M NaHCO3 + 10^−3^ M Na_2_SO_4_, at 25°C, by means of the potentiodynamic technique. The results indicate that the corrosion resistance of the FeCoC alloys depends on the carbon amount and the morphology of the phases present in the studied alloys.

## 1. Introduction

Cobalt is one of the first transition series of elements. It lays between Fe and Ni and close to Cu in the periodic table. In nature, it shows a strong spatial association with these metals. Cobalt is a critical metal and it has many strategic and irreplaceable industrial uses (supperalloys, magnets, corrosion- and wear-resistant alloys, high-speed steels, cemented carbides, diamond tool, etc.) [2–4]. Since cobalt shows great application potential, it has been widely studied.

This work is an academic study. It deals with the relation between the microstructure and electrochemical behavior of four iron-based FeCoC ternary alloys.

The solidification behavior of these alloys was studied in an earlier work [1]. This latter leads to the liquidus surface projection plot. In this paper, we undertake a study on electrochemical behavior of these alloys in nondeaerated solution of 10^−3^ M NaHCO_3_ + 10^−3^ M Na_2_SO_4_, at 25°C.

## 2. Experiment 

The studied alloys were arc melted in an argon gas atmosphere from pure elements (iron at 99.98 pct and cobalt at 99.5 pct from Aldrich Chemical Co.) and graphite. The solid-liquid and the solid-solid transformation temperatures were followed by a DTA-Netzsch 404S differential thermal analysis (cooling rate of 10 K/min) under argon atmosphere. The observation of the phases was performed using an optical microscope (ZEISSICM405) and a scanning electron microscope (SEM-JEOL). 

The electrochemical tests were conducted using a VoltaLAB PGZ301 potentiostat. The corrosive medium consisted of neutral aqueous solution containing 10^−3^ M NaHCO_3_ and 10^−3^ M Na_2_SO_4_. The polarisation curves are plotted in potentiodynamic mode. Potential was scanned from −0.8 V/SCE to +1 V/SCE in the direction of the increasing potentials at a scanning rate of 1 mV/s. Before each polarisation, the working electrodes were immersed in the test solution for 45 min. The electrochemical experiments were carried out at 25°C with agitation in presence of oxygen. 

## 3. Results and Discussion 

**Figure 1:**
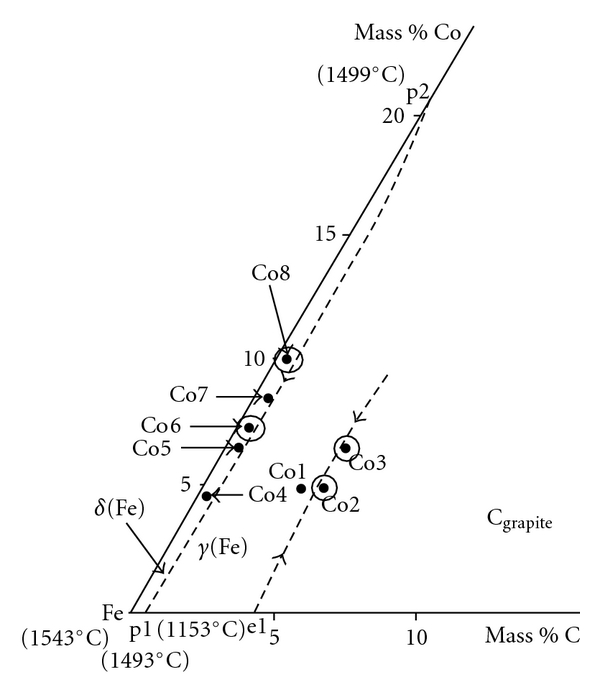
Liquidus surface projection of the Fe-Co-C system in the iron-rich corner (metastable system) [1] showing the studied alloys (encircled).

In an earlier study [1], the compilation of the differential thermal analysis results in relation to the observed microstructures as well as the analysis of different phases allows us to establish the solidification paths of the studied alloys. Thus, the primary crystallization phases and the univariant reactions have been identified. The obtained results are summarized in Table 1. The proposed liquidus surface projection of Fe-Co-C system in the iron-rich corner, presented in Figure 1, shows, for the studied alloys, the presence of two primary crystallization phases (*δ*(Fe) and graphite) as well as two univariante lines: eutectic L *↔γ*(Fe) + C_graphite_ and peritectic L + *δ*(Fe) *↔γ*(Fe). The studied alloys considered in this work are also shown in Figure 1 (encircled). 

**Figure 2:**
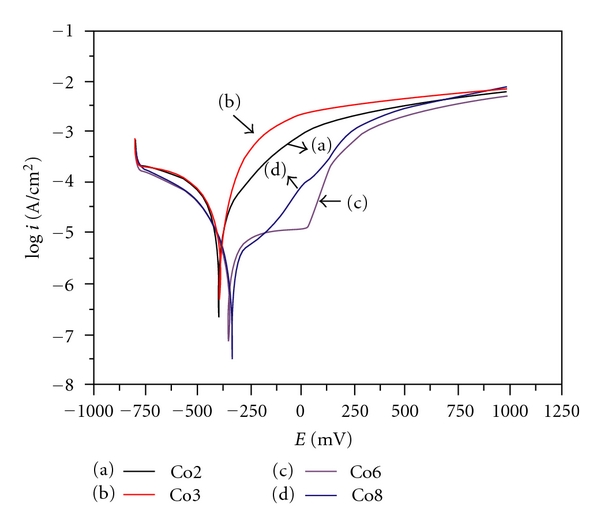
Potentiodynamic polarisation curves of Co2, Co3, Co6, and Co8 alloys in nondeaerated solution NaHCO_3_ 10^−3^ M + Na_2_SO_4_ 10^−3^ M, at 25°C.

Potentiodynamic polarisation curves of the studied alloys in nondeaerated solution containing 10^−3^ M NaHCO_3_ and 10^−3^ M Na_2_SO_4_ at 25°C are presented in Figure 2. The corresponding electrochemical parameters are given in Table 2. 

We gathered in Table 3 corrosion current densities (*i*_cor_) of the ternary FeCoC alloys with, respectively, the Fe/C ratio for each alloy. The results obtained for these alloys show that the corrosion current densities increase with the diminution of the Fe/C ratio.

Co6 and Co8 steels have a better corrosion resistance than Co3 and Co2 cast iron. This would be allotted to more important carbon content in cast iron. 

**Figure 3:**
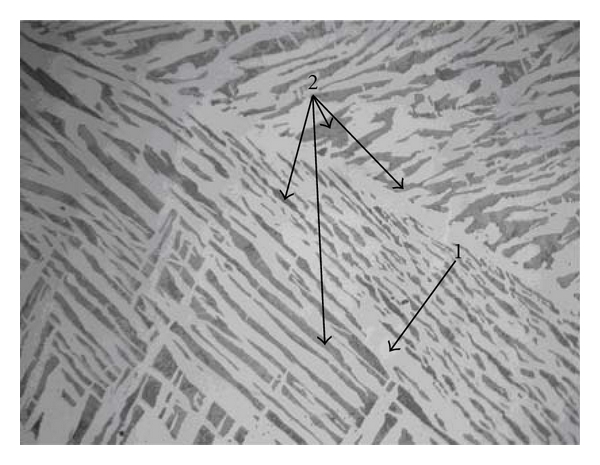
Optical micrograph (×200) showing the matrix (1) and pearlite (2).

**Figure 4:**
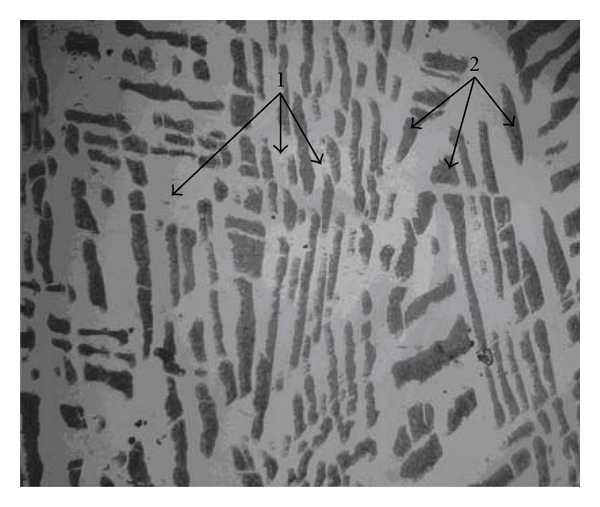
Co8 optical micrograph (×200) showing the matrix (1) and pearlite (2).

The Co8 alloy corrosion current density is slightly lower than that of Co6. For these two alloys, the effect of carbon and cobalt content does not appear. However, the microstructures of these alloys (Figures 3 and 4) present the same phases except that the pearlite structure is finer in Co6 alloy. This could explain the light increase of Co6 alloy corrosion current density. 

In fact, it was reported that pearlitic structures corrode faster than spheroidized materials and steels containing fine pearlite corrode rapidly than those with coarse pearlite. In addition, the degree of dispersion of the carbide is quantitatively characterized by the total amount of interfacial contact between the ferrite and cementite phases [5–8]. 

**Figure 5:**
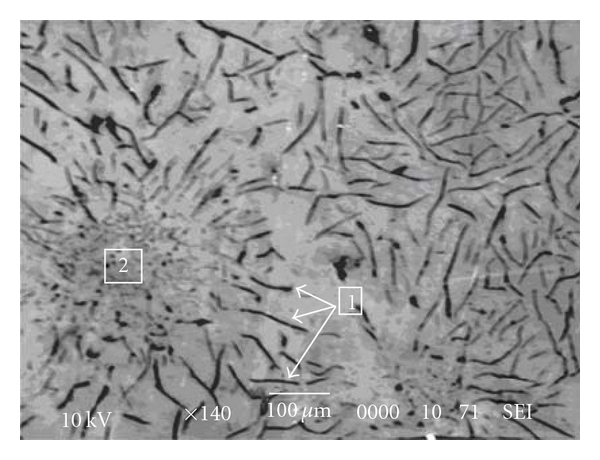
Co2 electron micrograph showing graphite (1) and *γ*Fe/C eutectic (2).

**Figure 6:**
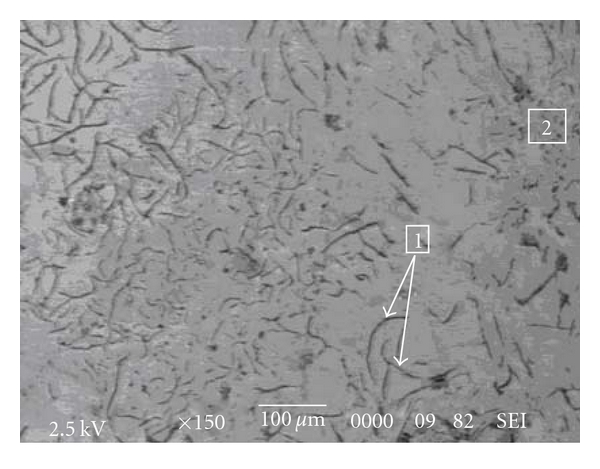
Co3 electron micrograph showing graphite (1) and *γ*Fe/C eutectic (2).

In addition, Co2 alloy is more resistant than Co3 alloy in the experimental conditions of this study. The examination of the microstructures of these two samples (Figures 5 and 6) shows that the structure of carbon graphite is finer in Co3 alloy. This would lead to an increase of corrosion current density [9]. 

## 4. Conclusion 

This work follows the study concerning the solidification behavior of iron-based FeCoC ternary alloys. The electrochemical behavior of some of these alloys is reported to solidification observed microstructures. 

The results show the presence of two primary crystallization phases (*δ*(Fe) and graphite) as well as two univariante lines: peritectic L + *δ*(Fe) *↔γ*(Fe) and eutectic L *↔γ*(Fe) + C_graphite_. 

The interpretation of the electrochemical results in relation with the observed microstructures leads to conclude that Co6 and Co8 steels have better corrosion resistant than Co2 and Co3 cast iron because of the more important carbon content in cast iron. Moreover, the corrosion current density increases with the decrease of in the Fe/C ratio. In addition, it was noted that the corrosion current density increases when the morphology is finer. 

## Figures and Tables

**Table 1:** Compositions, transformation temperatures, and solidification sequences of FeCoC studied alloys. (*Temperature not detected by our differential thermal analysis apparatus limited to temperature lower than 1550°C).

Alloy	Compositions (wt. %)			Temperatures/(°C)	Solidification sequences
	Fe	Co	C		
Co2	90.96	4.84	4.20	*	L *↔* C_graphite_
				1163	L *↔γ*(Fe)
				1150	L *↔γ*(Fe) + C_graphite_
				753	Pearlite
Co3	89.37	6.50	4.13	*	L *↔* C_graphite_
				1170	L *↔γ*(Fe)
				1153	L *↔γ*(Fe) + C_graphite_
				763	Pearlite
Co6	90.90	8.45	0.65	1496	L *↔δ*(Fe)
				1416	L + *δ*(Fe) *↔γ*(Fe)
				830	*γ*(Fe) *↔α*(Fe)
				756	Pearlite
Co8	89.52	10.00	0.48	1477	L *↔δ*(Fe)
				1463	L + *δ*(Fe) *↔γ*(Fe)
				812	*γ*(Fe) *↔α*(Fe)
				772	Pearlite

**Table 2:** Electrochemical parameters of FeCoC ternary alloys corrosion (immersed in 10^−3^ M NaHCO_3_ + 10^−3^ M Na_2_SO_4_, at 25°C).

Alloy	*E*_cor_/(mV/ECS)	*i*_cor_/(*μ*A/cm^2^)	*R*_*p*_/(kΩ·cm²)	*β*_*a*_/(mV/dec)	*β*_*c*_/(mV/dec)
Co2	−395	16.8	1.7	169	−179
Co3	−390	18.2	1.3	99	−178
Co6	−347	1.8	9.3	111	−82
Co8	−337	1.7	9.8	110	−88

**Table 3:** Variation of *i*_cor_ according to the Fe/C ratio.

Alloy	Co8	Co6	Co2	Co3
*i*_cor_ (*μ*A·cm^−2^)	1.7	1.8	16.8	18.2
Fe/C	186.5	184.5	21.66	21.64
